# Clean, cleaved surfaces of the photovoltaic perovskite

**DOI:** 10.1038/s41598-017-00799-0

**Published:** 2017-04-06

**Authors:** Márton Kollár, Luka Ćirić, J. Hugo Dil, Andrew Weber, Stefan Muff, Henrik M. Ronnow, Bálint Náfrádi, Benjamin Pierre Monnier, Jeremy Scott Luterbacher, László Forró, Endre Horváth

**Affiliations:** 1 Institute of Physics, Ecole Polytechnique Fédérale de Lausanne, CH-1015 Lausanne, Switzerland; 2 Swiss Light Source, Paul Scherrer Institute, CH-5232 Villigen, Switzerland; 3 Institute of Chemical Sciences and Engineering, Ecole Polytechnique Fédérale de Lausanne, CH-1015 Lausanne, Switzerland

## Abstract

The surface of a material is not only a window into its bulk physical properties, but also hosts unique phenomena important for understanding the properties of a solid as a whole. Surface sensitive techniques, like ARPES (Angle-resolved photoemission spectroscopy), STM (Scanning tunneling microscopy), AFM (Atomic force microscopy), pump-probe optical measurements etc. require flat, clean surfaces. These can be obtained by cleaving, which is usually possible for layered materials. Such measurements have proven their worth by providing valuable information about cuprate superconductors, graphene, transition metal dichalcogenides, topological insulators and many other novel materials. Unfortunately, this was so far not the case for the cubic, organo-metallic photovoltaic perovskite which morsels during the cleavage. Here we show a method which results in flat, clean surfaces of CH_3_NH_3_PbBr_3_ which allows surface sensitive measurements, badly needed for the understanding and further engineering of this material family.

## Introduction

The initial synthesis of the methylammonium lead iodide perovskite^1^ lead to interest in its optoelectronic properties^2^ and strongly correlated electron physics^3^. Such metal-organic halides have proven to be a breakthrough in photovoltaics. Since 2012 the solar cell efficiency has increased from, 11 to 22%^4–6^ and photo detection capabilities extended remarkably in both responsivity and spectral sensitivity^7, 8^. The progress in its microscopic understanding, however, is lagging behind. The application of the arsenal of surface sensitive techniques may help in this endeavour. Unfortunately, the surface of the majority of the as-grown 3D metal-organic halide crystals is degrading rapidly by absorbing water and losing the cation and the halide ions easily. The structure being 3D tetragonal/cubic near room temperature does not offer an easy cleavable surface. Here we show how to overcome this hurdle in the case of CH_3_NH_3_PbBr_3_ and demonstrate its success by high quality photoemission data in dark and under light illumination.

**Figure 1:**
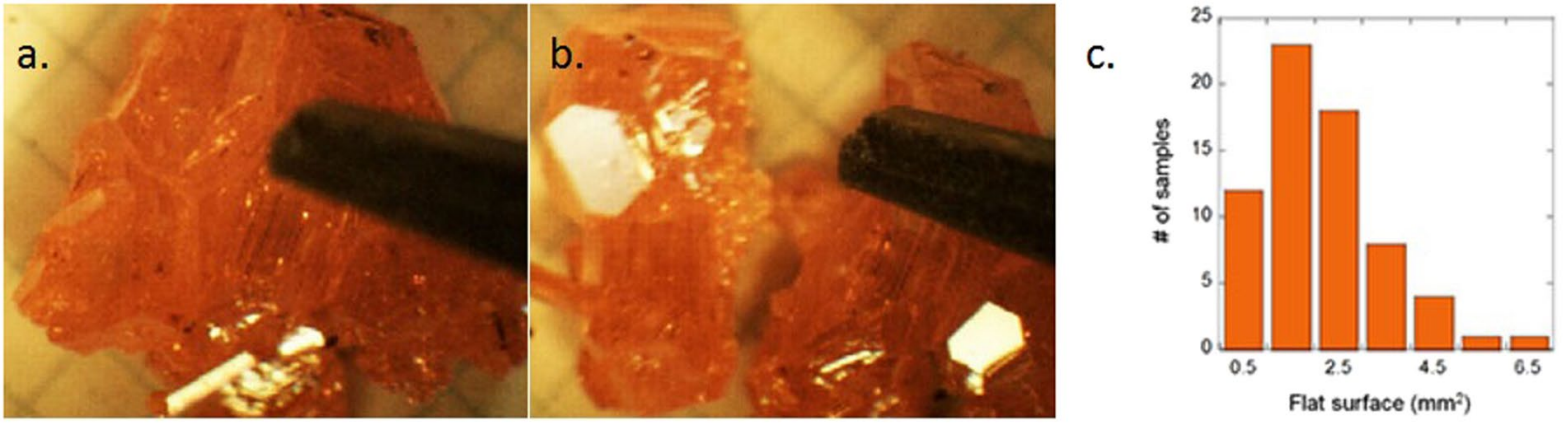
(**a**) Optical image of a cluster of CH_3_NH_3_PbBr_3_ single crystals mechanically stressed by the tip of a tweezers; (**b**) the broken-up cluster liberates a shiny, mirror-like surface previously embedded in the twinned crystals (the millimetre paper below the crystals show the scale); (**c**) Histogram of the cleaved, clean surface size of the CH_3_NH_3_PbBr_3_ single crystals.

Crystals of the methylammonium lead tribromide (CH_3_NH_3_PbBr_3_) were synthesized by solution growth^9^. The 3.3 mmol lead (II) acetate trihidrate (Pb(ac)_2_ × 3H_2_O, >99.9%) was reacted with 6 ml saturated HBr solution (48 wt% HBr in H_2_O). The formed PbBr_2_ precipitate is stable in the acidic solution. The respective amount (3.30 mmol) methylamine (CH_3_NH_2_) solution (40 wt% in H_2_O) was pipetted into the 5 °C ice cooled solution of PbBr_2_. The cold solution avoids the evaporation of methylamine during the exothermic reaction. Orange coloured microcrystallites of CH_3_NH_3_PbBr_3_ were formed immediately and settled down at the bottom of the vessel. In a temperature gradient of 15 °C in the acidic media, large (3–10 mm) intergrown orange, transparent crystals were formed in 7–10 days (Fig. 1a). Energy Dispersive X-ray Spectroscopy gave Pb:Br = 1:2.98 ± 0.02 atomic ratio confirming the stoichiometric composition of the samples.

After removing the crystals from the solution, they were dried on laboratory paper wipes at room temperature followed by 30 minutes air drying at 150 °C on a hot plate. During this heat treatment the colour of the crystals changed from light orange to dark orange-red. The colour change was reversible. Surprisingly, gravimetric analysis revealed that the as-synthesized crystals previously dried at room-temperature undergo an additional 1–5 wt% weight loss during the 150 °C drying. This unexpected observation raises the question what molecules leave from the crystal. Is the weight loss due to the decomposition of the CH_3_NH_3_PbBr_3_ or there are other volatile compounds present in the as-synthesized crystals? XRD and EDX measurements show that decomposition byproducts (e.g. PbBr_2_) are absent but temperature programmed desorption coupled with mass spectroscopy (TPD-MS) detect the solvent molecules (see Figs S7–S8 in SI). This shows that inclusions in the crystals, the “gallery spacing”, intra-crystalline voids between twin boundaries of the single crystals are filled with significant amount of solvent during the continuous cooling crystallization. To the best of our knowledge, this is the first observation of such high concentration of “mother liquor” in the single crystals and highlights the importance of careful degassing procedure, in order to obtain high quality and phase pure single crystals. We discovered, however, that the trapped mother liquor may have a favourable and practical side-effect in the creation of cleaved surfaces of metal halide perovskites.

We observed that by placing the crystals on the 150 °C preheated hot plate very often the intergrown crystals “exploded” due to the pressure generated by the trapped-in solvent. After such crystal breaking, large surfaces of the twin boundaries are revealed with a mirror-like, shiny appearance. Similar shiny surfaces could be obtained by applying a mechanical stress (by a tweezers, breaking lever in a photoemission UHV chamber) on a previously baked clusters of twinned crystals (Fig. 1b). Such a surface, which is highly advantageous in measurements requiring flat, non-contaminated surfaces attracted our attention. Figure 1c shows a histogram of the area of clean, flat surfaces after cleavage. One can see that the most frequent size is 1.5 mm^2^ but occasionally, exceptionally large surfaces of 6 mm^2^ could be obtained. It has to be mentioned that one can grow larger crystals of CH_3_NH_3_PbBr_3_ by using the inverse temperature crystallization strategy^10–12^ in N,N-dimethylformamide solution, but they do not cleave as well as those grown from HBr in H_2_O (see Fig. S1 in Supplementary Info (SI)). Apart from CH_3_NH_3_PbBr_3_, cleavage of CH_3_NH_3_PbI_3_ and CH_3_NH_3_PbCl_3_ can result in flat surfaces at the scale of the unit cell with however much lower success rate, (see Figs S2 and S3 in SI). One of the differences among the three compounds of the same family is their growth rate. CH_3_NH_3_PbBr_3_ has the fastest growth that results in much larger crystals having the agglomerates with large surfaces grown next to each other, which represent cleavage planes. It is likely that the pressure exerted by the trapped in solvent upon drying is beneficial for the nice cleaving. The characterization of such a surface is given below.

**Figure 2:**
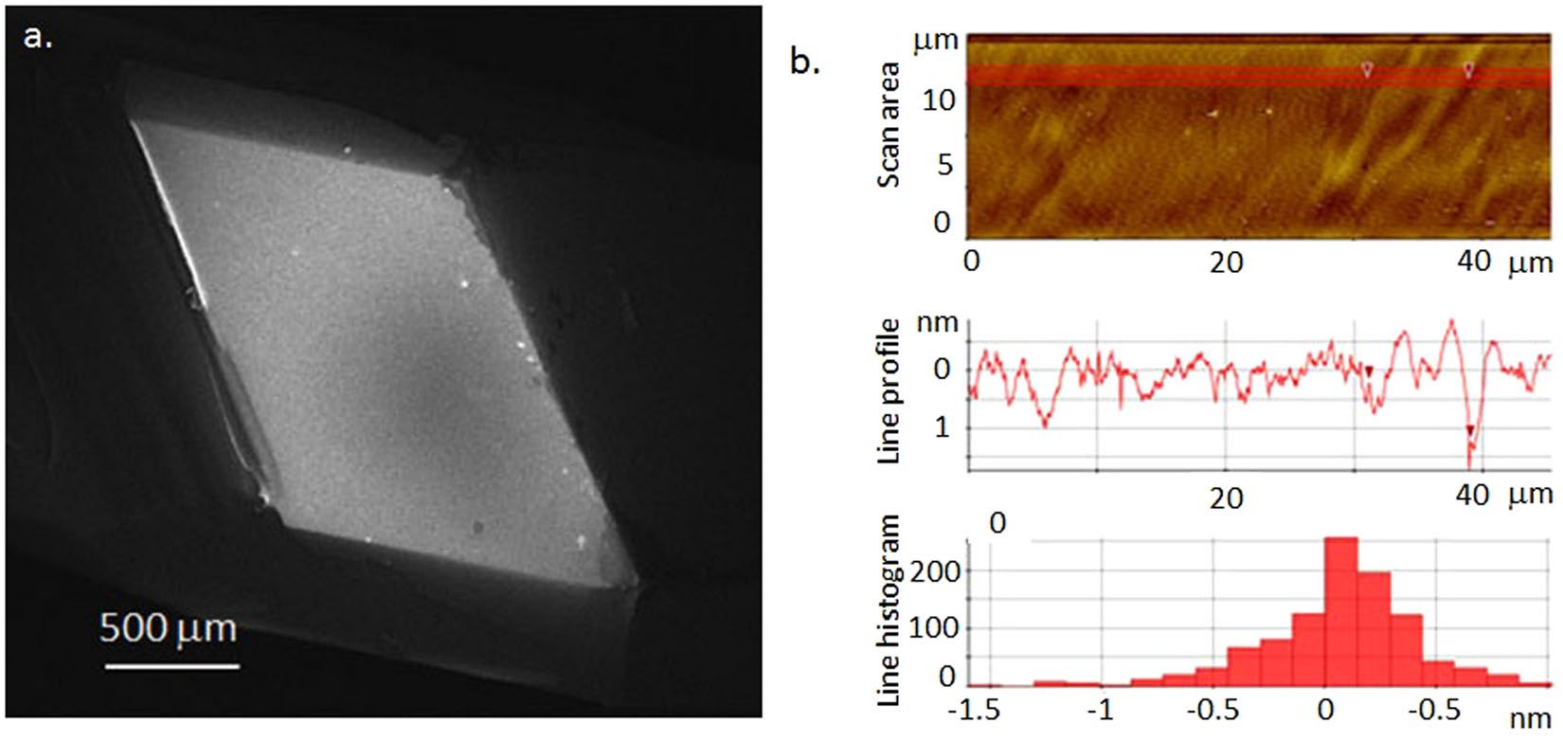
(**a**) SEM image of a freshly cleaved, large surface of CH_3_NH_3_PbBr_3_ single crystal. (**b**) AFM topographical scan of a representative area of the surface in Fig. 2a. The line scan shows at some places shallow pits.

The surface quality was checked by Scanning Electron Microscopy (SEM) and Atomic Force Microscopy (AFM). The SEM image of the cleaved surface (Fig. 2a) shows a flat, uniform surface over a millimetre wide area. Occasionally, one can find sub-micron sized cavities, which are packed with 20–50 nm size beads (see Supplementary Info, Fig. S4a in SI). Most probably these are secondary formations during the drying, when acidic lachrymation dissolves part of the surface and new formations appear. They do not influence any of the surface sensitive measurements. We have to note here, that the crystal is sensitive to the electron beam (3 kV), the surface is visibly damaged after 10 min of imaging (see Fig. S12b in SI).

Figure 2b shows an AFM topographic image of the freshly cleaved surface with flatness at the scale of the unit cell. The line-scans indicate at some places 1–5 nm deep and 2–5 nm wide pits on the surface. These pits are like inter-crystalline voids with the acidic liquor and they could be formed during the growth of the crystals or they are formed during the heat treatment of the samples. They as well do not represent any disturbance for spectroscopic measurements.

**Figure 3:**
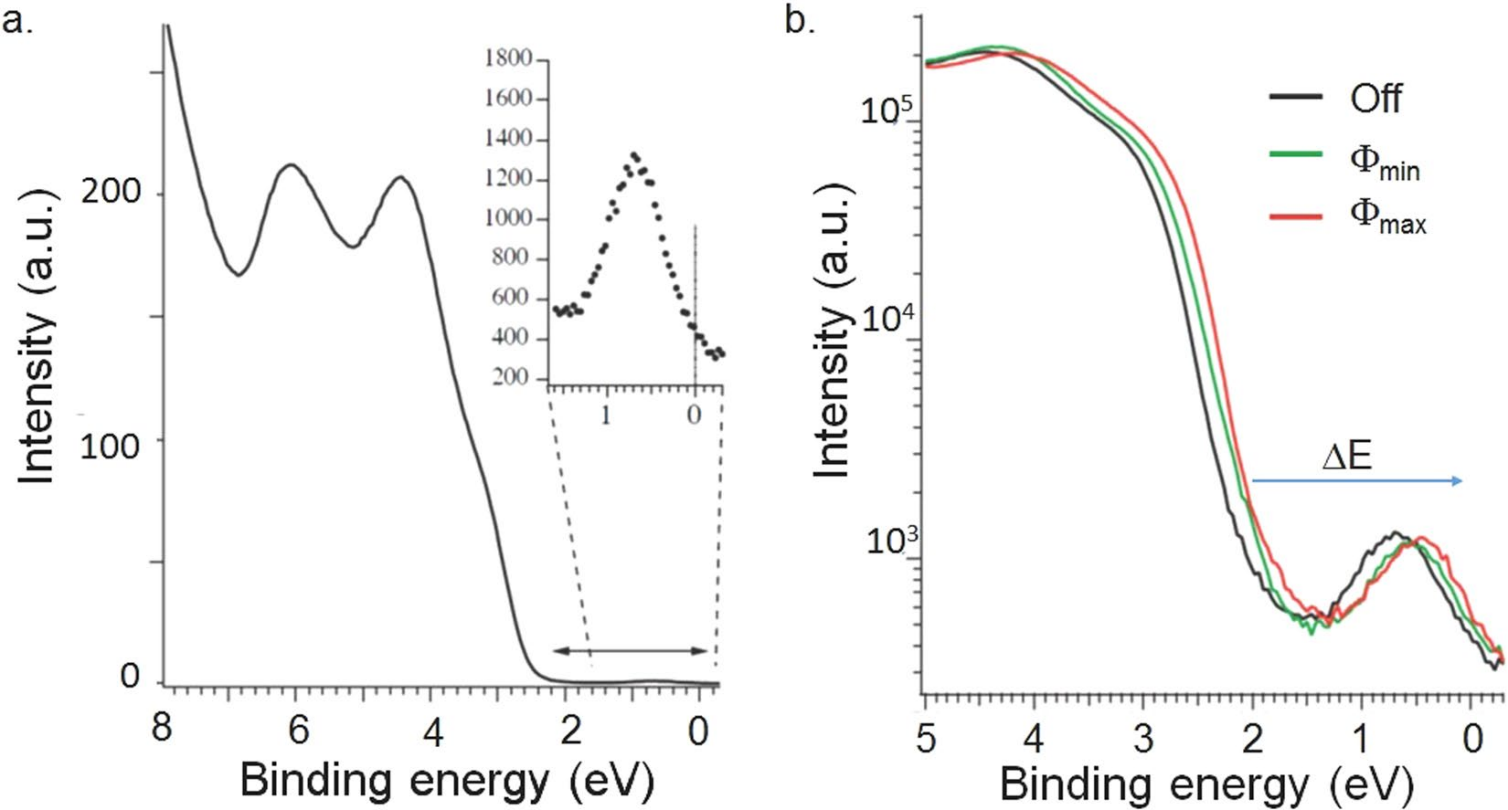
(**a**) Photoemission intensity as a function of binding energy obtained on *in-situ* cleaved CH_3_NH_3_PbBr_3_ single crystal surface. Beyond the LUMO levels at high binding energies, one can read clearly the 2.2 eV band gap and a well-defined excitation close to the Fermi level; (**b**) The semi-log plot reproduces figure a (black line) and to spectra measured under 1.5 mW (green line) and 4.5 mW (red line) white light illuminations. The density of states at the Fermi level increases with the light intensity.

Photoemission – ejecting electrons with photons to determine their energies – is a very important technique for probing electronic structures, but it is notoriously sensitive to surface effects, which explains the unfortunate scarcity of photoemission results on hybrid perovskites. Photoemission data obtained on an *in-situ*, mechanically cleaved crystal illustrate the importance of clean surface in spectroscopic measurements. The quality of the spectra are largely superior to those obtained on polycrystalline sample^13^ or on a randomly broken crystal surface^14^. The experiments were performed at room temperature using a hemispherical electron analyser in a spatially resolved mode. The photoelectrons were created by a defocused Helium gas-discharge UV-lamp beam instead of a typical focused synchrotron light to avoid beam damage (see Fig. S13 in SI). The full cleaved surface was illuminated to assure an equilibrium state. Figure 3(a) shows an overview of the valence band with two distinct features at 4.5 and 6 eV binding energy, resembling the LUMO and LUMO-1 levels of the material.

In Fig. 3a the photoelectron intensity rapidly drops towards the chemical potential and a clear band gap of 2.2 eV can be observed in the occupied states. However, when zooming into the region around the chemical potential a clear peak in the photoemission intensity at a binding energy of 0.8 eV (see inset) shows up unambiguously, well above the noise floor due to the high surface quality. Hints of such a feature were observed in a previous report on the methylammonium lead iodide and chloride sister materials^13^, attributed to excitonic bound states (“traps states”) originating from the *surface*, which are thought to limit electron-hole mobility and photovoltaic efficiency. The precise measurement of the peak position in our experiment suggests that its energy is most likely too high for being a Wannier exciton. We suspect that it is coming from the filling up of neutral impurity states by photoelectrons which could be important for the high efficiency of this perovskite family in photovoltaics^15^. But the understanding the precise nature of this peak will require further investigation with a variety of techniques in a well-calibrated environment.

The measurements in Fig. 3a and the black line in Fig. 3b were performed in the dark apart from the photons needed for measuring the spectral features. In order to study the response of this photovoltaic system we repeated the measurements with white light illumination of low (1.5 mW on sample) and high (4.5 mW) intensity. Remarkably, this leads to a shift of the bands and moves the peak feature closer to the chemical potential, thereby increasing the density of states at the Fermi level and thus increasing the local conductivity of the system needed for high photovoltaic performance. Switching off the light the initial state is recovered.

This report heralds that dedicated experiments in a well calibrated environment, on clean surfaces will be able to resolve many puzzles surrounding this intriguing class of materials. Our finding of *in-situ* cleavable high quality single crystals provides an essential first step for a whole class of experiment that will be able to pin down the mechanism of the photovoltaic activity of metal-organic perovskites and thereby lead the way for the design of even more efficient systems.

## Electronic supplementary material

Supplementary Information
