# Supplementary material for: Clean, cleaved surfaces of the photovoltaic perovskite
Source: Sci Rep. 2017 Apr 6;7:695. doi: 10.1038/s41598-017-00799-0 (PMC5429655; doi:10.1038/s41598-017-00799-0)
Supplement: Supplementary file 1 — Supplementary Information [file 41598_2017_799_MOESM1_ESM.pdf]

Clean, cleaved surfaces of the photovoltaic perovskite – supplementary information

Márton Kollár[1], Luka Ćirić[1], J. Hugo Dil[1, 2], Andrew Weber[1,2], Stefan Muff[1,2], Henrik M. Ronnow[1], Bálint Náfrádi[1], Benjamin Pierre Le Monnier[3], Jeremy Luterbacher[3], László Forró[1], Endre Horváth[1]
[1]Institute of Physics, Ecole Polytechnique Fédérale de Lausanne, CH-1015 Lausanne, Switzerland
[2]Swiss Light Source, Paul Scherrer Institute, CH-5232 Villigen, Switzerland
[3]Institute of Chemical Sciences and Engineering, Ecole Polytechnique Fédérale de Lausanne, CH-1015 Lausanne, Switzerland

1. Crystal growth and cleavage

In a different route by using N,N-dimethylformamide as solvent one can grow from saturated solution beautiful, large crystals of $CH_3NH_3PbBr_3$ using the inverse temperature crystallization strategy[1-3]. Under light it is translucent with an orange color (Fig S1). Careful temperature increase (2-4 °C/hour) will ensure continuous enlargement of individual single crystals. In contrary, implying sudden temperature increase (4-10 °C/hour) near the nucleation temperature will result in multiple seed formation and the growth of crystal intergrowth (see Fig 1Sa).

a.

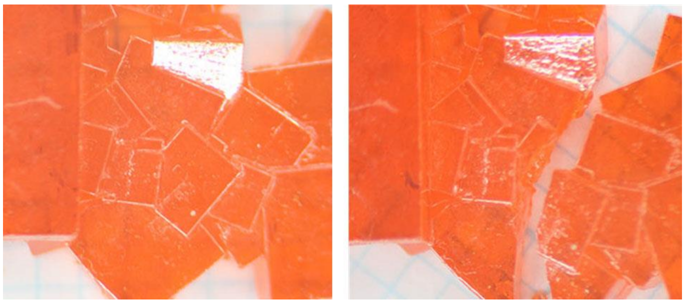

b.

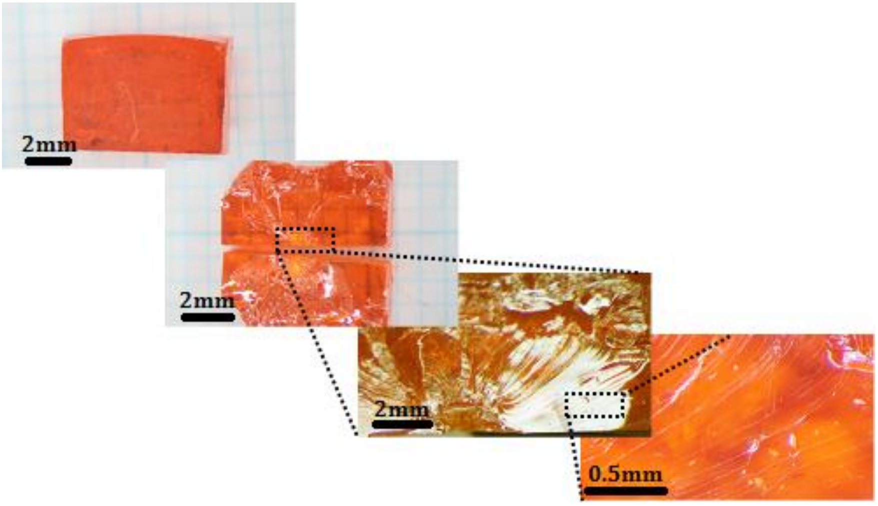

Figure S1. Illustration of various cleavage scenarios not resulting flat surfaces: a) different kind of single crystal agglomerates; b) cleaved surface of a big crystal of $CH_3NH_3PbBr_3$ (surface area 7.0 x 5.0 mm^2).

Interestingly, by applying a mechanical force these intergrowth $CH_3NH_3PbBr_3$ single crystals do not break along their grain boundaries. While breaking individual single crystals the middle of one face, at macroscopic scale it shows a regular break. However, by zooming of the broken surface one can realize that it has an un-even shell fracture, characteristic for glassy like minerals (see Fig S1b).

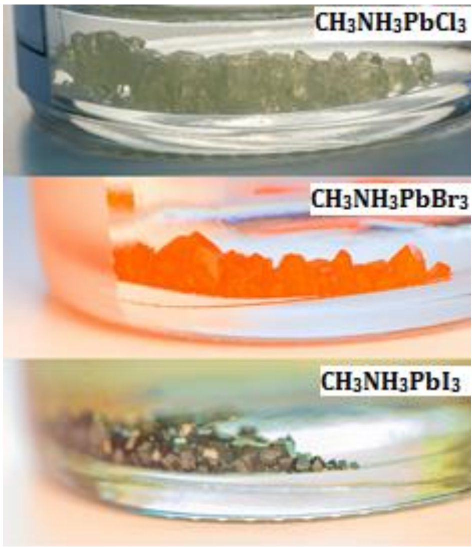

Figure S2a. Image of the growth, from the top to the bottom, $CH_3NH_3PbCl_3$, $CH_3NH_3PbBr_3$, and $CH_3NH_3PbI_3$ crystals. They all form agglomerates.

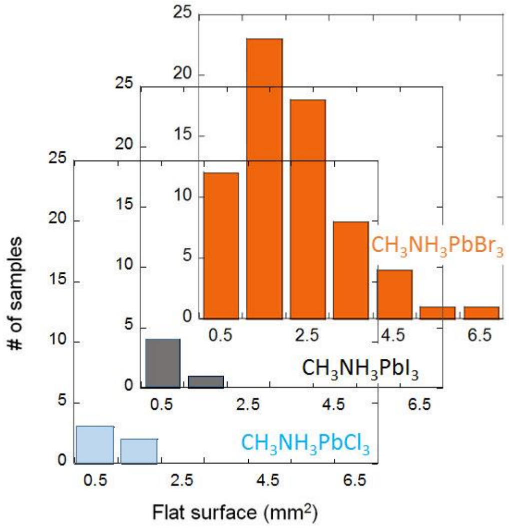

Figure S2b. Histograms of the area of cleaved surfaces of the three methylammonium lead halides.

The nice, atomically flat surfaces one can be obtained from the cluster of inter-grown $CH_3NH_3PbBr_3$ crystals formed in aqueous hydrobromic acid, as described in the main text. Such a growth can be achieved from the other two compounds of the same family

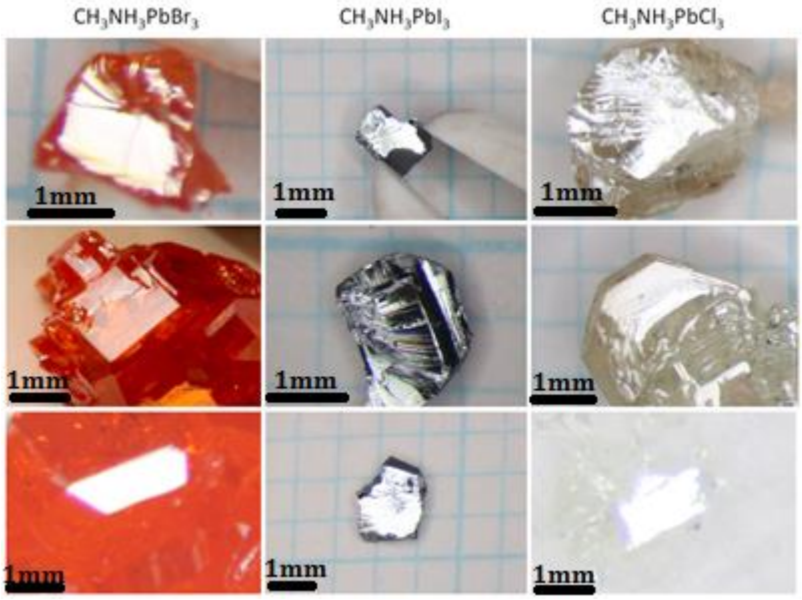

Figure S3. Characteristic images of the cleaved crystal surfaces from the clusters of intergrown crystal of $CH_3NH_3PbBr_3$, $CH_3NH_3PbI_3$, and $CH_3NH_3PbCl_3$.

as illustrated in Fig S2a. The fastest growth shows the $CH_3NH_3PbBr_3$ compound, which gives large crystals. Very often they agglomerate together, by sharing large surfaces,

which are easy planes for cleavage. Due to the fast growth, very often solvent is trapped in cavities of the agglomerates which also help the cleavage by heating up the crystals. Despite the fact that $CH_3NH_3PbI_3$ and $CH_3NH_3PbCl_3$ also form agglomerates, they can hardly give nice cleavage surfaces. A histogram of the surface area of the three compounds is given in Fig S2b. The number of broken crystals for each compound is very similar, but only the cases, when a nice surface could be identified are reported on the histogram. An illustration of the cleaved surfaces for the three compounds is shown in Figure S3.

2. X-ray diffraction, elemental analysis by X-ray fluorescence (XRF) and electron dispersive x-ray spectroscopies

The X-ray diffractogram was taken on an Empyrean system (Theta-Theta, 240mm) equipped with a PIXcel-1D detector, Bragg-Brentano beam optics. The cleaved surface of $CH_3NH_3PbBr_3$ crystal (Fig. S4), contains the characteristic lines of the oriented lattice planes of $CH_3NH_3PbBr_3$. The diffractogram is free from the characteristic lines of possible degradation products like $PbBr_2$.

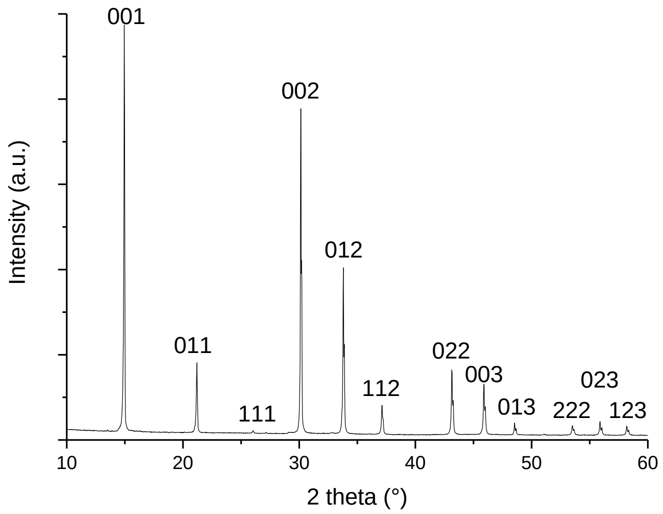

Figure S4. Characteristic X-ray diffraction pattern of a cleaved surface of $CH_3NH_3PbBr_3$ crystals grown in hydrobromic acid solution.

The X-ray fluorescence spectroscopy chemical mapping was done on Orbis PC Micro EDXRF analyzer system, equipped with a Rh micro-focus X-ray tube (50kV and 1mA) and X-ray multiple Optic turret with 30um poly-capillary X-Ray optics. The cleaved flat area (500 μm^2) of the $CH_3NH_3PbBr_3$ crystal grown in hydrobromic acid solution was examined. The data show that only Pb and Br are present in the crystal (beside the unfollowable light elements like C, N, O or H). No foreign elements as impurities have been identified.

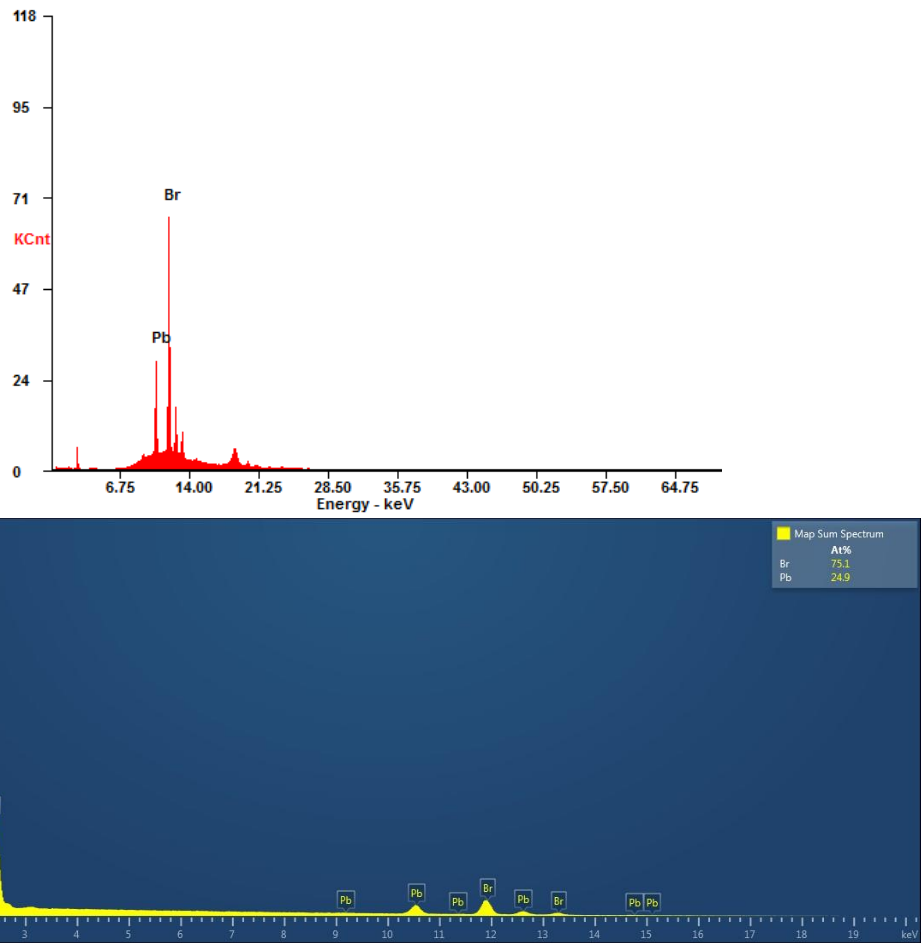

Figure S5. Elemental composition of a cleaved surface of $CH_3NH_3PbBr_3$ crystals by X-ray fluorescence spectroscopy (top panel) and EDX (bottom panel).

The energy-dispersive X-ray spectroscopy performed on $CH_3NH_3PbBr_3$ crystal gives a stoichiometry of 1:2.98 ± 0.03 for Pb an Br, which confirms the high quality of the sample.

3. Thermogravimetric analysis

The thermogravimetric analysis was done on a TGA4000 instrument from Perkin Elmer in N_2 atmosphere. The $CH_3NH_3PbBr_3$ crystals were dried on RT for two hours and were grinded in an agate mortar before the thermogravimetric analysis. During grinding the orange powder turned into a wet powder like consistency. We observed a significant loss of weight (1.15 wt%) in the initial isotherm step at 25 °C for 10 minutes. This we attribute to the evaporation of water, hydrobromic acid and acetic acid released from the crystal inclusions (see Figure S7 and corresponding paragraph). During the 10 °C/min heating to 200 °C the sample is loses weight continuously, with an increased rate between 110 °C and 130 °C. This corresponds to further water, hydrobromic acid and acetic acid evaporation.

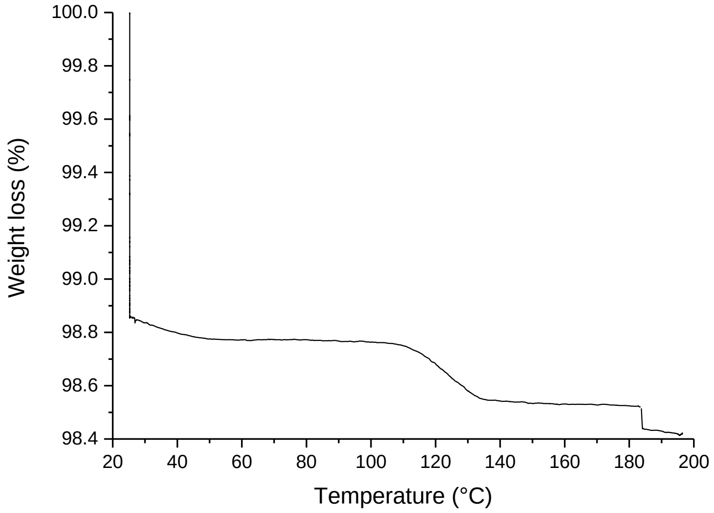

Figure S6. Thermogravimetric analysis of the powdered and room temperature dried $CH_3NH_3PbBr_3$ crystals.

Importantly, we observe a very sharp stair at 185 °C. This type of instantaneous weight loss appeared in several repeated measurements, when measuring the RT dried and non-grinded single crystals. It has been reported that some crystal inclusions might generate pressure 75 times that of atmospheric pressure. This extreme pressure accounts for their tendency to rupture during heat treatment[4]. Therefore, the sudden weight loss is very likely due to the loss of overpressurized liquid and/or gas trapped in the cavities. Under these circumstances „explosion"- type rupture happens, which manifests as a rapid weight loss in thermogravimetric analysis. Indirect prove of crystal explosion is that pieces of flown away $CH_3NH_3PbBr_3$ crystals have been found outside of the sample holder.

4. Temperature programmed desorption coupled with mass spectroscopy

Temperature Programmed Desorption (TPD) was performed on Micromeritics Autochem II 2920 equipped with Thermal Conductivity Detector (TCD) coupled to a MKS Cirrus 2 quadrupole Mass Spectrometer (MS). The carrier gas was He (Alphagaz, 99.9999%) at 30 mL/min STP. A quantity of 0.7 g of room temperature dried sample was crushed in a mortar and loaded in a quartz tube. The tube was plugged with quartz wool. The TPD was recorded from -20 to 100°C with 10 °C/min, and the maximum temperature was hold 60 minutes.

The results from the TPD-MS reveal that beside the water ($m/z^+=18$ and $m/z^+=17$) hydrobromic acid ($m/z^+=80$ and $m/z^+=82$) and acetic acid ($m/z^+=43$ and $m/z^+=45$) desorbs from the sample with a peak maximum at 70 °C.

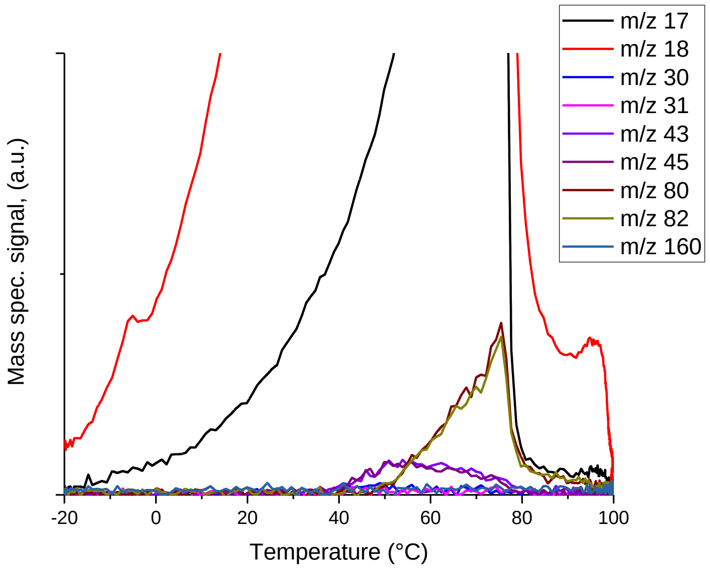

Figure S7. Analysis of the desorbing compounds from the room temperature dried and powdered $CH_3NH_3PbBr_3$ crystals by temperature programmed desorption combined with mass spectroscopy. The colours denote different masses, see details below.)

These measurements were repeated with the $CH_3NH_3PbCl_3$ crystals, where we can see the desorption of water, acetic acid and the HCl. In case of the $CH_3NH_3PbI_3$ crystals we had some technical limitations to see the I_2 signal ($m/z^+=254$), but we can follow the desorption of water, acetic acid and can see the iodine (I) signal ($m/z^+=172$).

If the measurement is done without crushing the crystals one can observe the “explosions” above 100°C where the gas and liquid trapped to inclusions release from the crystals (Fig. S8).

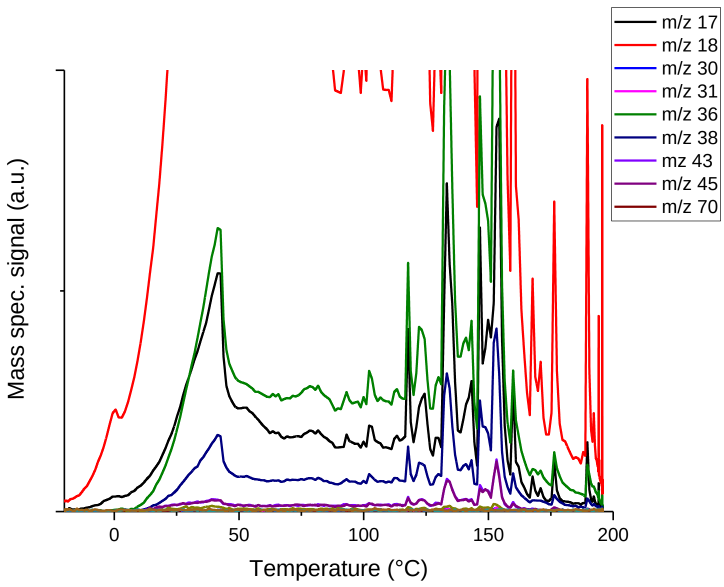

Figure S8. Analysis of the desorbing compounds from the room temperature dried $CH_3NH_3PbCl_3$ crystals (without grinding) by temperature programmed desorption combined with mass spectroscopy.

5. **Time lapse photographs and optical microscopy images**

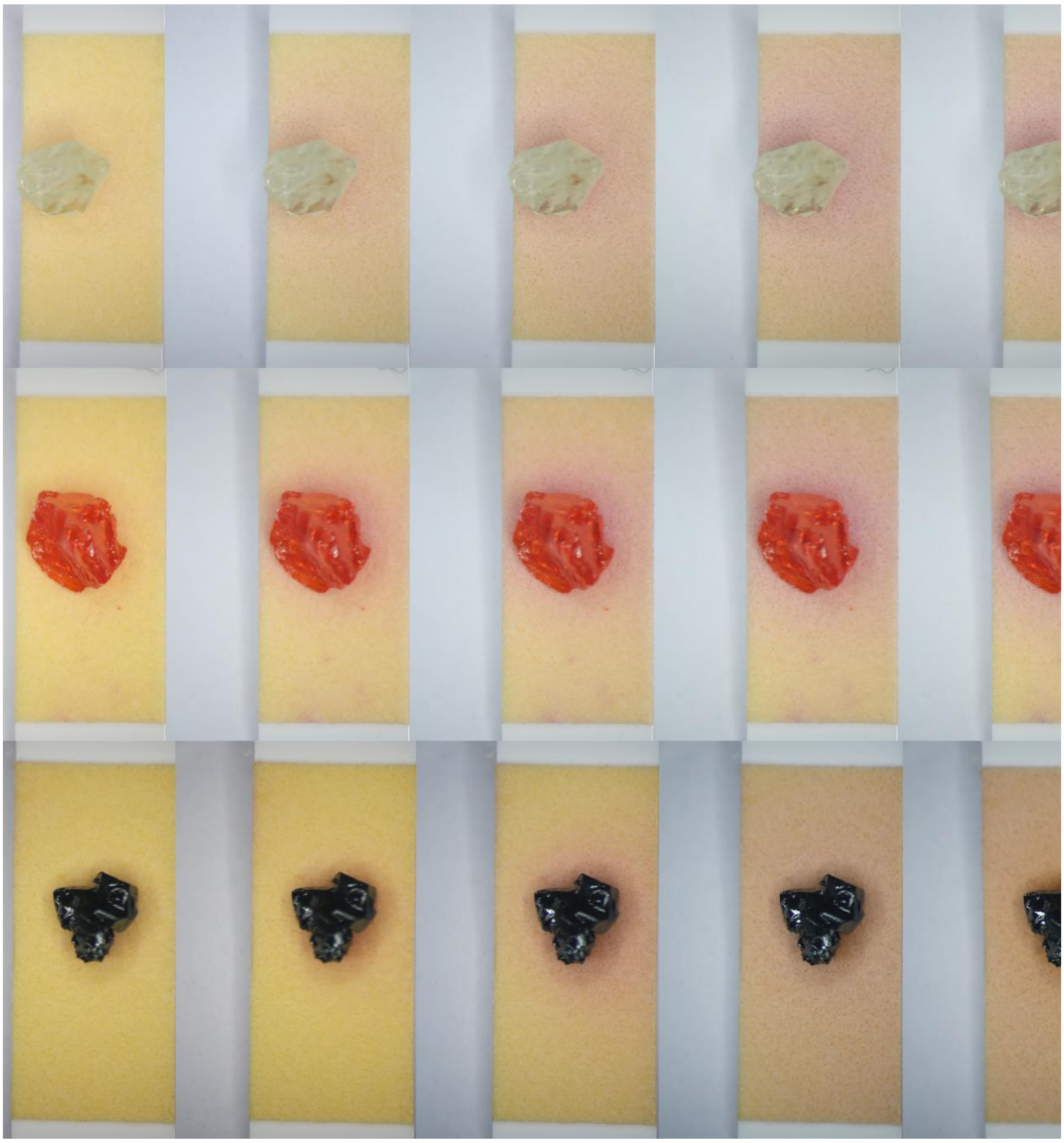

Figure S9 Time-lapse photographs of $CH_3NH_3PbCl_3$ (top row), $CH_3NH_3PbBr_3$ (middle row), $CH_3NH_3PbI_3$ (bottom row) single crystals grown in halide acid solutions and dried at RT for 2 hours. These crystals were then placed on pH-indicator strips (pH 0-2.5, Merck, 6 mm width). The colour change with time denotes the progressive release of respective halide acids.

Subsequent heat treatment of the previously room temperature dried crystals on a hot plate preheated to 150 °C induces visible yellow to red colour change of the pH-indicator strips (from left to right). This colour change is associated to the temperature induced breakage and release of respective halide acids from the two-phase negative crystal inclusions present in the crystals. The colour change has been observed 2 minutes after placing the crystals on the preheated hot plate.

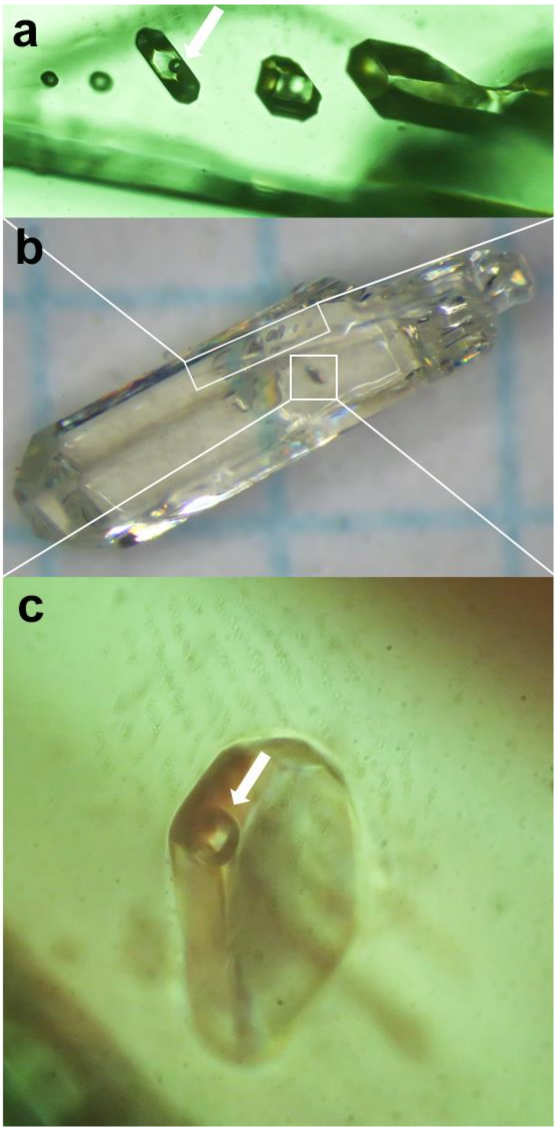

Figure S10 Optical microscopy image of $CH_3NH_3PbCl_3$ single crystals.

Optical microscopy image of $CH_3NH_3PbCl_3$ single crystals grown in hydrochloric acid solution (b). Several regular and irregular form cavities are observable (b). Higher magnification images reveal that these cavities are negative crystal inclusions of 10 to 200 µm in size. At room temperature, some of these cavities are two-phase inclusions, since gas bubbles can be clearly observed (a,c). Gas bubbles are marked with white arrows.

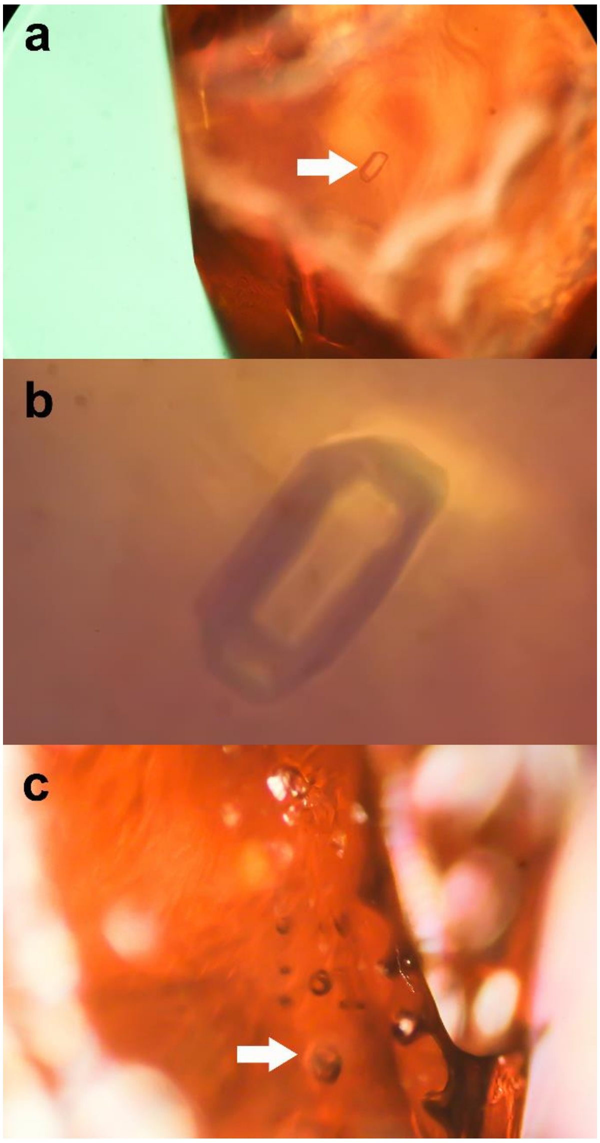

Figure S11 Optical microscopy image of $CH_3NH_3PbBr_3$ single crystals.

Optical microscopy image of $CH_3NH_3PbBr_3$ single crystals grown in hydrobromic acid solution (b). Several regular and irregular form cavities are observable (a-c). We analysed the content of the cavities by grinding the $CH_3NH_3PbBr_3$ crystals in a mortar. During grinding the orange powder showed wet powder like consistency. The resulting crushed powder was analysed with temperature programmed TG measurements (Figure S7 and paragraph). Water, hydrobromic acid and small amount of acetic acid desorbed from the powder. According to the TPD-MS results these are the main components filling up the cavities. These cavities might be syngeneic or epigenetic inclusions.

In many studies, macroscopic single crystal of perovskites are being used to reveal and understand the fascinating optoelectronic properties of hybrid halide perovskites. However, in some physical and chemical measurements the presence or absence of these inclusions could have cardinal importance, since they can severely affect the crystal quality, and ultimately lead to scattered data and false data interpretation. We emphasize, that the amount and type of these inclusions has to be studied more in detail.

6. Electron beam damage

The $CH_3NH_3PbBr_3$ crystal is more stable against humidity than the iodine containing sister compound, but it is quite sensitive to high energy particles like electrons or UV photons. In Fig. S12 the cleaved surface of the crystal was imaged in SEM. Fig. S12a shows that occasionally, here and there one can find shallow µm sized cavities, which are packed with 20-50 nm size beads. Most probably these are secondary formations during the drying which do not disturb the physical measurements. The shaded rectangle is coming from the e-beam damage of the crystal. This is emphasized in Fig. S12b, where one can notice the scanning paths as a darker regions on the image. The strongest lines correspond to 30 pA current (accelerating voltage 3 keV) over 60 s of exposure. It is very likely that the knock-on collisions of the electrons with the organic cation ($CH_3NH_3^+$) causes the damage.

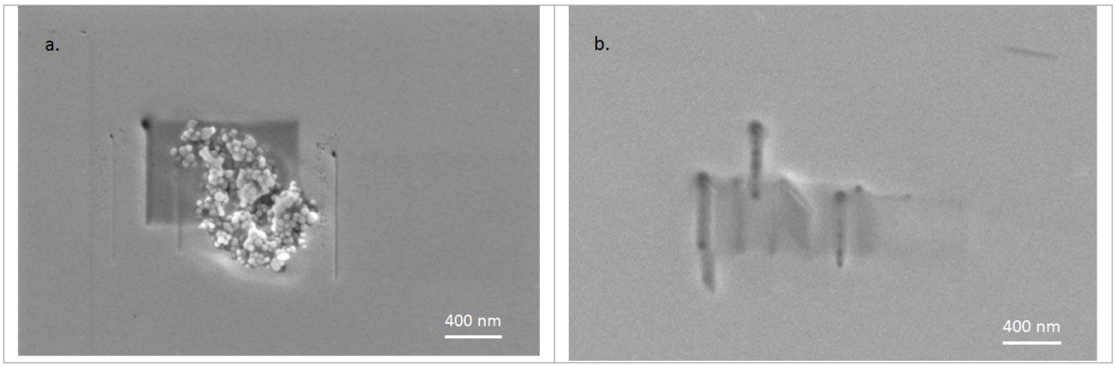

Figure S12 a) shows a flat, uniform surface over a millimetre wide area of $CH_3NH_3PbBr_3$. Sometimes, here and there one can find shallow µm sized cavities, which are packed with 20-50 nm size beads. Most probably these are secondary formations during the drying. The shaded are represents the electron beam damage, even better seen in b) with the imaging conditions of I = 30 pA, V= 3 keV, exposure time 60 s for the darkest lines.

7. High energy photon damage

One of the main problems in the study of organometallic photovoltaic perovskite by using photoemission spectroscopy is the radiation damage induced by the intense photon beam. Our initial studies using intense synchrotron radiation showed that the organic part of the material decomposes rapidly, rendering the obtained results non-representative for the material under investigation. Therefore, we decided to use a UV lamp with orders of magnitude lower flux density in order to obtain reliable valence band spectra. However, also under these softer conditions radiation damage induces a gradual change. This is illustrated in Figure S13 where we plot the time dependence of the obtained results. The measurements were performed by alternatively taking spectra with maximum intensity white light and without ambient light. Each spectrum took about 20 minutes and a clear shift of the features towards higher binding energy as a function of time can be observed. Over a period of about 2 hours this shift amounts to approximately 120meV.

The response to the white ambient light remains the same for all time steps, indicating that the photovoltaic properties remain intact. The observation that the radiation damage is relatively minor is also indicated by the fact, that the full valence band spectra after about 2 hours look almost identical to the ones for a fresh sample in the main text.

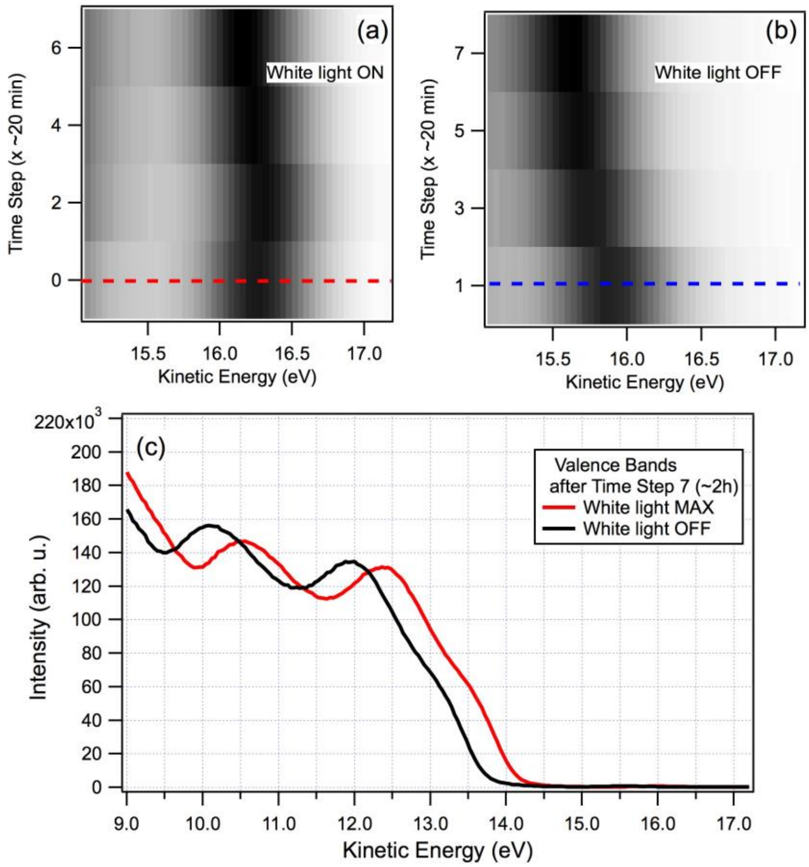

Figure S13 a,b) Time dependency of the exciton state close to the Fermi level with maximum intensity white light (a) and with lights off (b). The spectra were obtained alternating as indicated by the numbering. Each spectra took about 20 minutes. c) Full valence band spectrum obtained after the last spectrum of b).

Reference

1. M.I. Saidaminov et al. Chem. Comm. 51, 17658 (2015)
2. J.M. Kadro et al. Sci. Rep. 5, 11654 (2015)
3. M.I. Saidaminov et al. Nat. Commun, 6, 7586 (2015)
4. E. Roedder, Fluid Inclusions, Reviews in Mineralogy, Vol. 12, Mineralogical Society of America, Washington, DC, (1984)
